# Personalized 3D kidney model produced by rapid prototyping method and its usefulness in clinical applications

**DOI:** 10.1590/S1677-5538.IBJU.2018.0162

**Published:** 2018

**Authors:** Hakmin Lee, Ngoc Ha Nguyen, Sung Il Hwang, Hak Jong Lee, Sung Kyu Hong, Seok-Soo Byun

**Affiliations:** 1 Department of Urology, Seoul National University Bundang Hospital, Seongnam, Korea;; 2 Department of Urology, Cho Ray hospital, Ho Chi Minh city, Vietnam;; 3 Department of Radiology, Seoul National University Bundang Hospital, Seongnam, Korea

**Keywords:** Printing, Three-Dimensional, Kidney Neoplasms, Nephrectomy

## Abstract

**Background:**

Three-dimensional (3D) printing has been introduced as a novel technique to produce 3D objects. We tried to evaluate the clinical usefulness of 3D-printed renal model in performing partial nephrectomy (PN) and also in the education of medical students.

**Materials and Methods:**

We prospectively produced personalized renal models using 3D-printing methods from preoperative computed tomography (CT) images in a total of 10 patients. Two different groups (urologist and student group) appraised the clinical usefulness of 3D-renal models by answering questionnaires.

**Results:**

After application of 3D renal models, the urologist group gave highly positive responses in asking clinical usefulness of 3D-model among PN (understanding personal anatomy: 8.9 / 10, preoperative surgical planning: 8.2 / 10, intraoperative tumor localization: 8.4 / 10, plan for further utilization in future: 8.3 / 10, clinical usefulness in complete endophytic mass: 9.5 / 10). The student group located each renal tumor correctly in 47.3% when they solely interpreted the CT images. After the introduction of 3D-models, the rate of correct answers was significantly elevated to 70.0% (p < 0.001). The subjective difficulty level in localizing renal tumor was also significantly low (52% versus 27%, p < 0.001) when they utilized 3D-models.

**Conclusion:**

The personalized 3D renal model was revealed to significantly enhance the understanding of correct renal anatomy in patients with renal tumors in both urologist and student groups. These models can be useful for establishing the perioperative planning and also education program for medical students.

## INTRODUCTION

Rapid prototyping or additive manufacturing is a novel technology which can produce three dimensional (3D) objects from the computer-aided design data. This technology has been widely utilized by various industrial fields because it can easily and exquisitely produce 3D objects with very complex shapes from various raw materials such as silicon, plastic, and metal compared with the conventional methods (1). 3D printing also can manufacture various size of product from the smallest nano-particles to large buildings (2). In medical fields, 3D printing method drew attention several years ago. Until now, the most of medical application can be summarized into three fields: surgical planning, implant or tissue designing, and training models (3). For preoperative planning, the physicians produced the bony or vascular structures to understand the detailed anatomy and simulated the complicated surgical steps in advance by using the product (4).

Most of the contemporary medical imaging is based upon combinations of two-dimensional images. For performing a partial nephrectomy, it is utmost important to identify the exact location of tumor to minimize unnecessary dissection and bleeding (5). However, even in the experienced hands, the surgeons sometimes encounter problems in identifying the renal tumor particularly in small endophytic tumors. Furthermore, patient's position is another deteriorating factor. As the most of the renal surgery is performed in the flank position, the location of kidney tends to be rotated compared with the orientation of preoperative imaging studies.

We hypothesized that the stereoscopic three-dimensional personalized renal model can provide more detailed information during partial nephrectomy and also in training medical students. Therefore, we produced the personalized renal anatomy model by using 3D printing technique. We evaluated the clinical usefulness of renal model and also further implemented it in education for medical students. As the clinical usefulness can be subjective matter according to the clinician's ability to interpret the preoperative images, we tried to compare the outcomes separately in two independent groups with different experiences.

## MATERIALS AND METHODS

**Figure 1:**
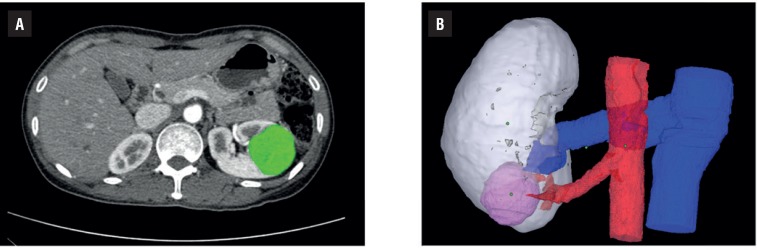
A) Preoperative images with additional processing for discrimination of tumor. B) The final 3D rendered images before printing.

**Figure 2:**
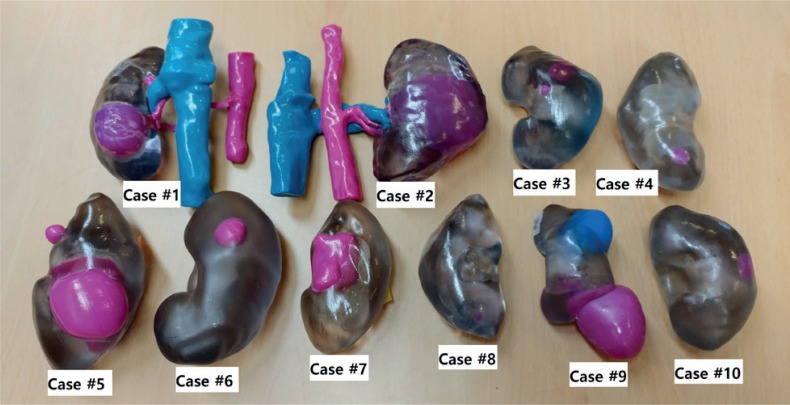
The produced 3D renal models for entire subjects.

After approval of our institutional ethical review boards, we prospectively enrolled 10 patients who were planned to receive robot-assisted partial nephrectomy for clinical T1 renal tumors by a single surgeon. The patient's clinical and pathologic information were retrieved from our institutional electronic medical recording system. The pathologic staging, cancer subtyping, and histologic grading were performed as previously described (6). Preoperative evaluation included multi-dimensional abdominal CT and chest radiography (or CT). After the patients were scheduled for surgery, personalized 3D kidney models were produced using the patient's preoperative CT images. The patient's DICOM data was extracted and our uro-radiologist processed the DICOM images by adding the outlines of tumors and renal parenchyma (Figure-1). Subsequently, the DICOM data was converted into STL files. Those processes were performed by using two softwares (Compact View III Ver. 1.03. Optimum Solution, Korea, Blender v2.76. Blender foundation, Amsterdam, NL). From these STL files, 3D models were created by using the Object 260 Connex 3 (Stratasys, Eden Prairie, MN, USA) with assistance of 3D manufacturing company (Optimum solution, Korea). The 3D model was built by using photopolymer of two colours (transparent: renal parenchyma, red: tumor) (Figure-2). Two different questionnaires were developed to evaluate the clinical usefulness of 3D renal model and each questionnaire was asked to be completed by two independent groups (urologist group, student group). The urologist group included the surgical team who participated in each patient's surgery (one attending surgeon, one board-certified urologist as first assistant, and one resident). And the student group included a group of twenty medical students who had poor prior experiences on interpreting CT scan. The student group was questioned to locate the tumor solely using CT images and questioned again with help with 3D renal model to answer the correct tumor location. They also were asked to appraise the clinical usefulness of each modality (CT only, CT + 3D renal model) in terms of tumor localization and anatomic understanding for each case. The questionnaire for urologist group consisted of five questions for clinical usefulness of 3D models in understanding for anatomy and preparing the preoperative surgical planning and localizing the tumor during surgery and about willingness to utilize the 3D model in future. The independent t-test and chi-square test were performed to compare the perioperative characteristics between groups. SPSS software package (SPSS 19.0, Chicago, IL, USA) was utilized for statistical analyses. All p-values presented were two-sided and p < 0.05 was considered statistically significant.

## RESULTS

The overall clinical and pathologic characteristics were summarized in Table-1. Median age was 58.0 [interquartile range (IQR) 39.0 - 59.0] years and median tumor diameter was 18.5 (IQR 12.0 - 52.5) millimeters. The production cost for each renal model was $650 each. The urologist group gave highly positive answers for all end-points regarding the clinical usefulness of 3D renal model (understanding personal anatomy: 8.9 / 10, preoperative surgical planning: 8.2 / 10, intraoperative tumor localization: 8.4 / 10, plan for further utilization in future: 8.3 / 10, clinical usefulness in complete endophytic mass: 9.5 / 10). When the student group tried to evaluate the exact location of renal tumor only by using preoperative CT scan, they answered correctly only in 47.3%. However, with assistance of 3D-models, the rate of correct answers was significantly elevated up to 70.0% (p < 0.001). On asking the subjective difficulty level in evaluating the location and shape of the renal tumor, they reported difficulty to be significantly low (52% versus 27%, p < 0.001) with the use of 3D renal models. The urologist group also appraised the clinical usefulness of 3D renal model very highly in terms of locating the tumor (8.8 of 10) and understand the associated anatomy (8.9 of 10).

**Table 1:** Baseline characteristics of all patients.

Case number	#1	#2	#3	#4	#5	#6	#7	#8	#9	#10
Age	59	55	60	40	58	36	39	59	61	58
Sex	F	F	F	F	M	M	M	F	M	F
Laterality	Right	Left	Left	Left	Left	Right	Left	Left	Left	Left
Tumor size	40 mm	55mm	14 / 11 mm	14 mm	86 / 11mm	22mm	50mm	8 mm	60 / 32 mm	15mm
Location	Low pole	Mid pole	Upper / Mid pole	Low pole	Mid / Upper pole	Upper pole	Mid pole	Low pole	Low / Upper pole	Mid pole
Shape	Exophytic	Exophytic	Mesophytic	Endophytic	Mesophytic	Mesophytic	Exophytic	Endophytic	Endophytic	Endophytic

## DISCUSSION

In the present study, we tried to evaluate the clinical impact of 3D personalized renal anatomic model. As the urologist group has high ability to interpret and understand the renal anatomy from CT scan, we tried to evaluate how much the 3D model could enhance the perceptive function in the student group. From our results, we found that student group could locate the renal tumor more correctly with the help of 3D renal model. Moreover, the urologist and student group both appraised the clinical value of 3D anatomic model very highly in our study. Initially, medical applications of the 3D printing technology were focused on producing patient-tailored implant materials and / or helping preoperative surgical planning (7–9). As the cranio-facial or maxilla-facial surgery have high needs in understanding the complex bony anatomy, 3D printing was reported to be beneficial in those surgeries. D'Urso et al. utilized 3D printing method to produce patients-customized implants for defects of cranial bone and reported that the 3D printing was easier and cheaper and better for making patient-tailored implants which can provide more cosmetic effects (7). Faber et al. tested the 3D printed dental models to establish the preoperative surgical plan and also made target teeth-specific customized implant materials (8). They concluded that the anatomic models and implant materials produced from 3D printing were very useful compared with the conventional way of making implant materials. Another study by Guarino et al. produced the 3D pediatric spine and pelvic models to evaluate the clinical usefulness (9). They suggested that the application of 3D models can be beneficial for preoperative planning, intra-surgical navigation and also in patient counseling. However, most of early studies about medical applications of 3D printing were focused in the bony structure, because bones are quite easy to differentiate from other soft tissue during the process of images in producing 3D model. But the 3D printing technique can be also benefit not only 3D reproduction of bony anatomy but also for soft tissue anatomy, since various materials such as plastic, silicon and even chocolate, can be utilized for 3D printing (10). Several study groups tried to make individualized auricular prostheses by using 3D printing methods (11, 12). They utilized the images of contralateral auricle to create the 3D shape of target prosthesis. Oshiro et al. utilized the 3D printed liver model to determine optimal resection line before surgery and simulate hepatectomy (13). Apart from the reconstruction of the individual anatomy, several groups tried to utilize the 3D printing technique to develop training models for medical training (14–17). White et al. created 3D urinary tract model for ureteroscopic training (14). They utilized the post-contrast images to identify the urinary tract and reproduced it as artificial urinary tract by 3D printing method. They concluded that their urinary tract model appeared to be very useful for the endourological training. Bruyere et al. also developed a percutaneous nephrolithotomy trainer using 3D printing technique (15). They put a stone inside the trainer and let the trainee perform percutaneous nephrolithotomy in a more realistic way. Although their training models were quite pioneering, there have been no further publications after the initial reports of primitive training models. In our present study, the student group which consisted of medical students, showed significantly better understanding of the renal anatomy when using the 3D renal model. We believe that our 3D renal model can have extra value in educating the medical student.

Prior to our study, other groups also tried to create 3D kidney model with renal tumor for patients who were going to be treated by partial nephrectomy. Silberstein created five 3D renal units with tumors by 3D printing method (18). They reported that the patients and their family verbally expressed improved understandings about their disease status and treatment modalities. Bernhard et al. performed a prospective study and created 3D-renal model in seven patients before partial nephrectomy (19). They focused on whether the 3D renal model can enhance the patient's understandings about the renal anatomy and upcoming surgery. From the response of patients upon their questionnaires, they concluded that the 3D renal model facilitated patient's understandings about the surgery significantly with high level of satisfaction. On the other hand, the present study sought to evaluate the clinical usefulness of 3D renal model both in the experienced and unexperienced medical personnel. Not only the unexperienced group but also experienced group highly appraised the 3D renal model in understanding the correct anatomy and also establishing preoperative surgical plan.

The present study is not free from certain limitations. First of all, our study is limited from its small sample size. Due to the relatively high production cost, it was difficult to produce 3D renal models in many patients. However, as the production cost is getting cheaper and cheaper, the accessibility for the 3D printing will increase in future. Second, there is a possibility of selection bias toward to include patients with complex shape of tumor in the present study. Nonetheless, we believe that our endeavors for image processing and 3D producing are clinically valuable in developing virtual or augmented reality-based surgical platform in the future.

## CONCLUSIONS

Personalized 3D renal model revealed to be clinically useful in understanding renal anatomy better in both urologist and student groups. These 3D renal models can help surgeons to establish the surgical plan and also can be good education materials for medical students.
